# Injury History and Mental Health Indicators in Young Soccer Players: A Cross-Sectional Study

**DOI:** 10.3390/medicina62040667

**Published:** 2026-04-01

**Authors:** Alejo García-Naveira, Carmen Cerezuela Díaz, Laura Gil-Caselles, Aurelio Olmedilla-Zafra

**Affiliations:** 1 Department Psychology, University Villanueva, 28034 Madrid, Spain; alejo.garcian@villanueva.edu; 2 Department Psychology, University of Murcia, 30100 Murcia, Spain; carmencerezuelad@um.es; 3 Research Group HUMSE, Faculty of Sports Sciences, University of Murcia, 30720 Murcia, Spain; 4 Research Group HUMSE, Department of Personality, Evaluation, and Psychological Treatment, Faculty of Psychology, University of Murcia, 30100 Murcia, Spain; olmedilla@um.es

**Keywords:** adolescents, injury risk, psychological factors, sport psychology

## Abstract

*Background and Objectives*: The relationship between mental health and sports injuries has become increasingly important in youth soccer, due to developmental changes in this population, the high demands of training, and the competitive pressures of sport. This cross-sectional study examined the association between injury history (no injuries, 1–2, >2 injuries), mental health indicators (anxiety, stress, depression), and differences by sex, competitive category, and playing position. *Materials and Methods*: 146 soccer players (79 males, 67 females; ages 12–30; mean age = 16.65, SD = 2.34 years) from youth and senior categories of a professional club in Spain completed the STAI-T (trait anxiety), DASS-21 (state anxiety, stress, depression), sociodemographic and sports-related variables (gender, sports category, playing position), and self-reported injury history: no injuries (*n* = 39), 1–2 injuries (n = 80), >2 injuries (n = 27). The statistical analyses performed were one-way ANOVA (ηp^2^), χ^2^ tests, and Games-Howell post hoc tests. *Results*: 73.3% of the players reported ≥1 injury (54.8% 1–2 injuries; 18.5% >2), with no differences by gender, position, or category (χ^2^ range: *p* > 0.05). The ANOVA revealed significant differences for trait anxiety (F(2, 143) = 3.68, *p* = 0.029, ηp^2^ = 0.049; small-to-moderate), and state anxiety (F(2, 143) = 4.63, *p* = 0.014, ηp^2^ = 0.061; moderate). No effects were found for stress/depression (*p* > 0.12). The post hoc test (Games-Howell) indicates that the group with no injuries showed significantly lower trait anxiety (*p* = 0.038, d = 0.33) vs. 1–2 injuries, and state anxiety (*p* = 0.012, d = 0.70) vs. >2 injuries. Stress and depression showed a non-significant upward trend. *Conclusions*: A greater history of injuries is associated with higher levels of anxiety in youth soccer players. The findings suggest routine assessment of anxiety and training in emotional self-regulation for injury prevention and rehabilitation. Longitudinal studies are needed to clarify the bidirectional relationship.

## 1. Introduction

In the context of soccer, it is particularly important to analyze the factors associated with the occurrence of injuries due to their high prevalence and incidence. In recent studies (2018–2024) conducted with young soccer players from European academies and Spanish youth leagues, the injury incidence can vary widely depending on exposure, ranging from 0.5 to 45 injuries per 1000 h of training and competition over the course of a full official season [1,2]. Among young competitive players, between 34% and 66.5% suffer at least one injury during the season [3,4].

These data take on special importance considering that injuries are common in competitive sports and lead to interruptions in athletic activity that can cause pain, emotional distress, frustration, loss of confidence, difficulties in daily life, and uncertainty about the future [5,6,7]. These authors suggest that, consequently, injuries affect not only physical health but also mental health, negatively impacting the athlete’s psychological well-being and quality of life.

Traditionally, sports injuries have been explained primarily by biomechanical factors (e.g., muscle imbalances, joint misalignments, mobility limitations), physiological factors (e.g., accumulated fatigue, insufficient recovery, strength deficits), and sports-related factors (e.g., high training load, excessive competitive volume, early specialization) [8,9,10]. However, there is growing evidence that psychological factors and mental health also influence both the onset and recovery from injuries. In this regard, the relationship between mental health and injury has been described as bidirectional: certain psychological indicators can increase vulnerability to injury, while the injury itself can significantly and persistently affect the athlete’s psychological well-being [11,12].

Within this framework, Andersen and Williams’ Psychological Model of Sports Injuries [13,14,15] conceptualizes injury as the result of the interaction between individual factors (e.g., personality traits, anxiety, coping skills), contextual factors (e.g., competitive pressure, social support, training conditions), and physiological and emotional responses to stress. This interaction influences both the risk of injury and recovery processes, as well as vulnerability to future relapses [16,17,18].

This perspective is particularly relevant for children, adolescents, and young athletes, given their developmental stage, characterized by growth processes and hormonal, physical, psychological, social, and athletic changes, which can increase their vulnerability to stress and, consequently, their risk of injury [19,20,21].

Previous evidence has linked sports injuries to anxiety, stress, and depression in the adult population. In longitudinal studies of elite athletes, those who suffer recurrent injuries show significant increases in depressive symptoms (OR = 1.8–2.3) and anxiety (r = 0.32–0.45) during periods of competitive inactivity [22,23,24]. Similar patterns have been observed in injured adolescent athletes undergoing rehabilitation, with high competitive anxiety and difficulties regulating stress [25].

In young soccer players, recent studies have shown that a higher frequency and severity of injuries is associated with higher levels of anxiety, stress, and depressive symptoms, highlighting anxiety trait as a particularly sensitive variable in those who have suffered at least one injury [4,26]. These findings suggest that trait anxiety and state anxiety symptoms may constitute sensitive markers of psychological vulnerability in youth soccer players.

In young soccer players, Sánchez-Ruiz et al. [4] identify trait anxiety as a sensitive marker (≥1 injury), a finding confirmed by Moreno-Fenoll et al. [26], who found that a higher frequency and severity of injuries is associated with higher levels of anxiety, stress, and depression.

In youth categories, injury risk analysis must also take into account variables specific to the sporting context, such as age/category and playing position. In youth soccer programs, the incidence of injuries tends to increase as the competitive level rises (IRR = 1.4–2.1), likely due to greater physical and psychological demands [27]. Prospective studies in youth soccer indicate that, with age, muscle injuries and joint sprains increase, as does the risk of overuse or growth-related injuries [3,4,28]. Likewise, playing position influences exposure and injury profile: field players, especially midfielders and wingers, tend to have higher injury rates (HR = 1.6) due to greater locomotor and participation demands, while goalkeepers tend to show a lower overall incidence, though with a specific profile associated with explosive actions, falls, and impacts [27,29,30].

Recent literature on the bidirectional relationship between mental health and sports injuries suggests that injury recurrence, rather than a single isolated event, is associated with progressively greater psychological distress, fear of reinjury, and increased vulnerability. On this basis, previous studies in athletes have differentiated between no injuries, moderate injury history (one–two injuries), and repeated injury history (more than two injuries) as qualitatively different risk profiles [22,23,24]. Therefore, it is particularly important to distinguish between players with no recent injury history, players with a moderate injury history (one–two), and players with a pattern of repeated injuries (more than two), as the latter may present a higher psychological risk profile and require specific interventions.

Consequently, the objective of this cross-sectional study was to examine the association between injury history (no injuries, 1–2, >2 injuries), mental health indicators (anxiety, stress, depression), and differences by sex, competitive category, and playing position in youth soccer players. Specifically, the aim is to determine whether there are distinct profiles among uninjured players, players with a moderate history of injuries, and players with a history of repeated injuries based on their levels of anxiety, stress, and depression, and to verify whether these profiles differ according to gender, sports category, and playing position. Thus, the study seeks to fill the existing gap in the literature regarding combined profiles of injury history and mental health in youth soccer, incorporating both individual and contextual factors.

Consistent with previous evidence and the Psychological Model of Sports Injuries, the following research hypotheses are proposed:

**H1.**  *Players with a moderate injury history (one–two) and a repeated injury history (more than two) will exhibit significantly higher levels of anxiety, stress, and depression compared to injury-free players, with an expected increasing gradient based on the number of injuries*.

**H2.**  *Differences in mental health indicators based on injury history (no injuries, one–two, more than two) will vary according to sociodemographic and sports-related variables (gender, competitive level, and playing position), such that higher-level categories and certain field positions will exhibit specific patterns of psychological risk*.

## 2. Materials and Methods

### 2.1. Design

Following the methodological classification by Ato et al. [31], a non-experimental study with an associative-comparative, cross-sectional design was conducted to analyze the relationship between variables and compare differences between groups without manipulation or random assignment, using descriptive variables to classify participants and collecting data at a single point in time. The study report followed the STROBE guidelines for observational research [32].

### 2.2. Participant

The final sample consisted of 146 soccer players from various teams within a professional club in Spain, aged 12–30 years (M = 16.65, SD = 2.34). Non-probabilistic convenience sampling was used. Inclusion criteria were: (a) belonging to one of the club’s youth or senior teams during the current season; (b) regular participation in training and competition (at least two weekly sessions plus the league match); and (c) not having an acute injury that prevented usual participation at the time of assessment. Players who did not fully complete the questionnaires or who presented comprehension difficulties were excluded. The initial pool comprised 165 players, of whom 19 were excluded for these reasons. Detailed distributions by competitive category and sex are presented in Table 1, Table 2 and Table 3.

For the purposes of this study, “sports injury” was defined as any physical injury occurring during training or competition that resulted in the interruption or modification of regular sports participation, for example, total absence or limited participation in at least one training session or official match [2,3,4]. This definition was explained to the players before they completed the questionnaire on injury history.

The wide age range (12–30 years) reflects the club’s structure, which integrates youth categories (Under-12, Under-14, Under-16) and a Senior team within the same organizational framework. Since age is strongly correlated with competitive category, the analysis was conducted using the sports category as the primary variable, which allowed for grouping players into homogeneous competitive levels consistent with standard practice in youth soccer. However, in the descriptive analyses, age means by category are reported to facilitate interpretation of the heterogeneity.

The players completed between three and four training sessions per week, each lasting approximately 90 min, in addition to participating in league matches on the weekend. The assessment process was conducted by a licensed psychologist specializing in sports psychology with over six years of professional experience, under the supervision and guidance of a senior sports psychologist with over 25 years of professional experience.

### 2.3. Instruments

A questionnaire designed specifically for this study was used to collect data on the participants’ sociodemographic and athletic characteristics, such as competitive level, usual position on the field, and history of injuries during the previous season, including their presence and number.

Depression, Anxiety, and Stress Scale-21 (DASS-21). To assess the participants’ recent emotional state, the validated Spanish version of the DASS-21 [33] was used, based on the original instrument [34]. This self-report questionnaire consists of 21 items distributed across three subscales of 7 items each, which assess depression, anxiety, and stress using a 4-point Likert-type scale (0 = “never,” 3 = “almost always”). The depression subscale explores symptoms of sadness, lack of motivation, and decreased interest or pleasure; the anxiety subscale measures tension, fear, and anxiety-related physiological arousal; and the stress subscale assesses irritability, difficulty relaxing, and feelings of being overwhelmed. Participants responded based on how they had felt during the past week, in accordance with the instrument’s original instructions, which allows for the capture of recent emotional state. The DASS-21 exhibits high internal consistency (α > 0.80) and adequate convergent and discriminant validity [33]. In the sample of this study, the reliability coefficient was α = 0.90.

State-Trait Anxiety Inventory (STAI-T). To assess anxiety as a trait, the STAI-T subscale was used, consisting of 20 items with responses on a 4-point Likert scale [35], adapted into Spanish [36]. This self-report questionnaire measures a person’s stable tendency to perceive situations as threatening and to habitually react with anxiety. In other words, it quantifies the individual’s predisposition to experience chronic anxiety, regardless of momentary circumstances. Previous studies show that the STAI-T has high internal consistency (α > 0.86) and good validity in the Spanish population [36]; in the sample of this study, an α of 0.93 was obtained.

The combination of the STAI-T subscale and the DASS-21 allows for the assessment, respectively, of the stable predisposition to experience anxiety and recent symptoms of anxiety, stress, and depression. This integrated approach is particularly useful in the athletic population, as it enables a distinction between dispositional vulnerability and emotional distress associated with the competitive context and recent traumatic experiences [19,22,24].

### 2.4. Procedure

In the initial phase, the club’s sporting director and the head of the Psychological Support Service were contacted to present the project. Subsequently, the coaches were informed about the procedure and the conditions for administering the assessment instruments.

Data collection was conducted during the second half of the competitive season, from February to April, coinciding with a period in which match load and prior exposure to potential injuries were already representative of the sporting season. The instruments were administered in paper-and-pencil format, in group settings, without the presence of the coach, in the locker rooms of the teams that met the necessary assessment conditions, including adequate temperature, lighting, noise level, and available space. The administration process was adapted to each team’s training schedule.

The questionnaire on injury history included items on the presence or absence of injuries in the previous season and the total number of injuries, following the operational definition of sports injury described above. Sociodemographic and sport-related questions covered age, sex, competitive category, and primary playing position.

The assessment was carried out by the sport psychologist responsible for the evaluation, who was not involved in technical decision making or in the club’s medical staff, thereby reducing the risk of bias related to athletic performance. Participants received standardized instructions on how to complete the questionnaires, and any questions regarding comprehension were addressed before the assessment began. The evaluator had no detailed prior information about the participants’ individual injury history beyond what the players themselves reported in the questionnaire; therefore, the evaluator was considered blinded to the specific hypotheses of the study.

The study was conducted in accordance with the principles of the Declaration of Helsinki [37,38,39] and the Ethical Standards in Sport and Exercise Science Research [40]. In addition, the study was approved by the Research Ethics Committee of the University of Murcia (Spain) (reference: CEI-4734-2023; approval date: 20 July 2023). Participation was entirely voluntary, and the participants received no financial compensation for their collaboration. Written informed consent was obtained from all participants and, in the case of minors, also from their parents, guardians, or legal representatives.

### 2.5. Statistical Analysis

Statistical analyses were performed using IBM SPSS (version 21). First, descriptive statistics, including means and standard deviations, were calculated for anxiety, stress, and depression according to injury history. The assumption of normality was assessed using the Kolmogorov–Smirnov test, together with inspection of histograms and skewness and kurtosis values, revealing an acceptably normal distribution for the psychological variables across the different groups.

To examine group differences according to the number of injuries (0, 1–2, and >2), a one-way analysis of variance (ANOVA) was conducted. Because Levene’s test indicated a violation of the homogeneity of variance assumption (*p* < 0.05) for some variables, the Games–Howell procedure was used for post hoc comparisons. In addition to *p* values, effect sizes were calculated, including partial eta squared (ηp^2^) for the main ANOVA effects and Cohen’s d for pairwise comparisons, in accordance with common recommendations for the use of parametric tests and effect sizes in observational research [32,40].

The association between number of injuries and categorical variables, specifically sex, competitive category, and playing position, was examined using chi-square tests. The assumption of expected cell frequencies (≥5) was checked, and cases in which this criterion was not met were identified and considered a limitation in the interpretation of the results. For all analyses, the significance level was set at *p* < 0.05, and multiple-comparison procedures based on Games–Howell were applied to control for the increased risk of Type I error in the post hoc tests.

## 3. Results

Of the total sample (N = 146), 73.3% reported at least one injury (54.8%: 1–2 injuries; 18.5%: >2 injuries; 26.7%: 0 injuries). See Table 1.

### 3.1. Distribution of Injury History According to Sociodemographic and Sport-Related Variables

Table 1, Table 2 and Table 3 present the distribution of the number of injuries (0, 1–2, >2) according to sex, playing position, and competitive category. No statistically significant differences were found for any variable (χ^2^ ranges: *p* > 0.05), although the analyses by category and playing position showed violations of the expected frequency assumption (see table notes).

### 3.2. Mental Health Indicators According to Injury History

Table 4 presents the descriptive statistics for the psychological indicators across injury history groups. A pattern of progressive increase was observed in all four indicators as the number of injuries increased.

The one-way ANOVA revealed significant differences in trait anxiety, F(2, 143) = 3.68, *p* = 0.029, ηp^2^ = 0.049 (small to moderate), and anxiety (DASS-21), F(2, 143) = 4.63, *p* = 0.014, ηp^2^ = 0.061 (moderate). No significant differences were found for stress, F(2, 143) = 2.11, *p* = 0.129, or depression, F(2, 143) = 0.98, *p* = 0.377.

Levene’s test indicated a violation of the homogeneity of variances assumption (*p* < 0.05) for both anxiety measures; therefore, the Games–Howell test was used for post hoc comparisons (see Table 5).

**Figure 1:**
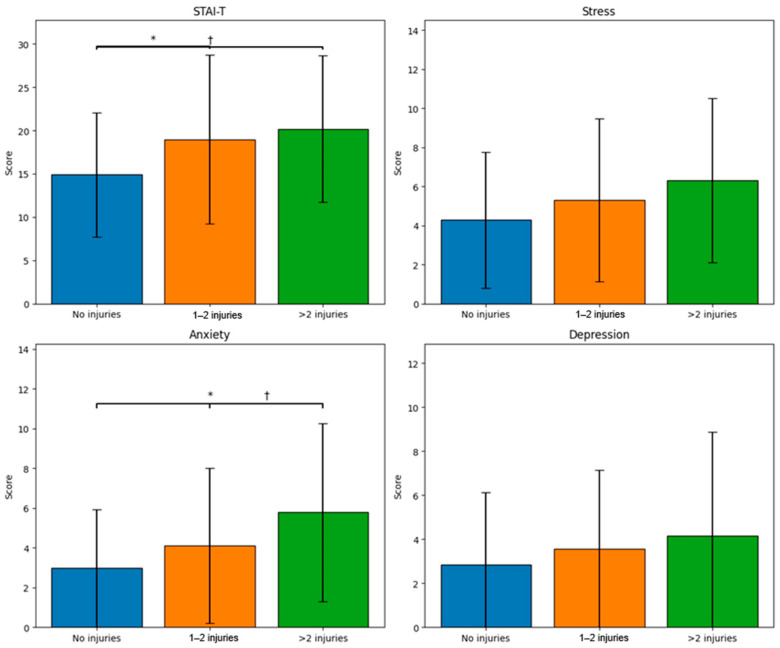
Mental health indicators according to the number of injuries in young soccer players. Note. Bars represent mean scores for each psychological variable across injury groups. * *p* < 0.05; † *p* < 0.10. STAI-T = Trait Anxiety; DASS-21 = Depression, Anxiety, and Stress Scales.

Taken together, these findings suggest that injury history is mainly associated with indicators of anxiety, while stress and depression do not appear to vary significantly depending on the number of injuries, although an increase can be seen as the frequency of injuries increases (see Figure 1).

## 4. Discussion

The present study examined the relationship between sports injury history and mental health indicators in young soccer players, while also considering sociodemographic and sport-related variables. The results showed that most participants (73.3%) reported at least one injury during the season, with one to two injuries being the most common pattern. This high prevalence is consistent with previous literature in youth soccer, which has reported variable but substantial injury rates among adolescents [1,2,3,4], and it reinforces the importance of addressing mental health in this context.

Regarding mental health indicators, the results partially support H1, showing that a greater injury history was mainly associated with higher levels of anxiety, whereas stress and depression showed similar trends but did not reach statistical significance. Findings in this direction have been reported in adult athletes [22,23,24] and young soccer players [4,26].

Even so, the most consistent findings were observed for the anxiety measures. Both Trait Anxiety (STAI-T) and anxiety (DASS-21) showed a progressive increase as the number of injuries increased, reaching significant differences between players with no injuries and those with one or more injury episodes. The STAI-T, which measures a person’s general tendency to perceive situations as threatening and to respond with anxiety, in this case reflects a greater overall predisposition to experience anxiety in players with 1–2 and more than 2 injuries [35,36].

In turn, the anxiety subscale of the DASS-21, which assesses recent symptoms such as tension, fear, and physiological arousal, showed that players with more than 2 injuries experienced greater emotional distress at the time of assessment than those with no injuries or with one to two injuries [33,34]. The convergence of both instruments suggests that injury history is associated with both higher levels of trait anxiety and greater recent anxiety symptoms, which may reflect sustained psychological vulnerability in young soccer players with a higher frequency of injuries.

This pattern suggests that anxiety may be a particularly sensitive variable in the relationship between injury history and psychological distress in young soccer players, as proposed by Andersen and Williams’ Psychological Model of Sport Injury [13,14,15], in which individual factors and emotional responses to stress interact to influence both the occurrence of and recovery from injuries. However, given the cross-sectional nature of the design, it is not possible to establish the directionality of this relationship. Anxiety may increase vulnerability to injury, but the experience of repeated injuries may itself contribute to higher anxiety levels, pointing to a possible bidirectional relationship between mental health and sports injuries [11,12].

In the case of stress and depression, the differences between groups did not reach statistical significance, despite a descriptive trend toward higher scores as the number of injuries increased. Therefore, these findings should not be interpreted as evidence of a meaningful effect, but rather as results that do not fully replicate the more consistent associations between injuries and depressive symptoms reported in previous studies [22,23,24]. This discrepancy may be due, among other factors, to the limited sample size, the heterogeneity of the sample, and the cross-sectional, self-report nature of the assessment.

The results also highlight the importance of considering contextual and sociodemographic variables when interpreting psychological profiles, in line with H2. Although no significant differences were observed by sex, competitive category, or playing position in the injury frequency analyses, the literature indicates that these variables do influence exposure to injury and the type of injury sustained [3,4,28,29,30]. The absence of significant differences in this sample may be explained by the limited size of some subgroups, such as goalkeepers and players in the U14 category, as well as by the high heterogeneity in participants’ sporting experience.

Likewise, with respect to sex, the distribution of injuries was relatively homogeneous between males and females. However, variables such as age and competitive experience may influence exposure and injury type, consistent with previous studies showing an increased risk of injury as physical and sport-specific demands rise in higher competitive categories [27,28,30].

On the other hand, although no statistically significant differences were observed according to competitive category, the obtained value was close to the conventional threshold for significance. In this regard, the U14 category stands out, as 71.4% of players reported no injuries, which could be related to lower physical and competitive demands at these ages, as suggested by previous research [3,4].

## 5. Limitations

The present study has several limitations that should be considered when interpreting the results. First, the cross-sectional design prevents the establishment of causal relationships between injury history and mental health indicators; therefore, the findings should be understood in associative terms. It is not possible to determine whether anxiety acts as a predisposing factor for injury or whether it is a consequence of injury. Second, information on injury history was obtained through self-report, which may introduce recall bias and variability in the subjective perception of injury, especially in developmental categories. In addition, relevant variables such as injury severity, type of injury, and time elapsed since the most recent injury were not considered, despite their important role in shaping the psychological response. Third, the sample showed substantial heterogeneity in age (12–30 years) and competitive category, together with small subgroup sizes (goalkeepers, n = 14; U14 category, n = 7). This limited the statistical power to detect differences in contextual variables such as playing position and competitive category. In particular, 41.7% of the χ^2^ cells had expected frequencies below 5, requiring cautious interpretation. In addition, the sample was drawn from a single professional club, which restricts the generalizability of the findings to other competitive contexts and developmental structures. Finally, although validated instruments were used (STAI-T α = 0.93; DASS-21 α = 0.90) and effect sizes were calculated (ηp^2^ = 0.049–0.061), the exclusively self-reported assessment, without clinical interviews or objective measures, limited the depth of the psychological analysis. The wide confidence intervals observed in small subgroups also requires cautious interpretation.

## 6. Practical Applications

Despite the limitations noted above, the findings of this study have relevant practical implications for youth soccer. First, they highlight the importance of an interdisciplinary approach to the prevention, identification, and management of mental health indicators associated with sports injuries and players’ psychological well-being. In this regard, the medical and coaching staff of youth clubs could systematically incorporate the assessment of anxiety, both trait and state, in players with a history of one to two injuries, and especially in those with more than two, using brief instruments such as the STAI-T and the DASS-21 to identify profiles with greater psychological vulnerability. These assessments could be conducted at the beginning of the season, mid-season, and after an injury.

Second, once these at-risk profiles have been identified, specific interventions can be implemented from an interdisciplinary perspective and integrated into injury prevention and rehabilitation programs, with the aim of reducing fear of reinjury and facilitating a gradual and safe return to competition. Within this framework, the interdisciplinary team would include the sport psychologist, responsible for assessment and cognitive-behavioral intervention, such as coping skills training, cognitive restructuring, and emotional regulation techniques; the sports physician, responsible for the objective evaluation and monitoring of injuries; the strength and conditioning coach, responsible for designing and supervising load progression and return-to-play reconditioning; the head coach, who can identify relevant behavioral signs in the sport context, such as increasing irritability, unexplained fatigue, or avoidance of physical contact, and facilitate the practical implementation of interventions; the podiatrist, who assesses and helps prevent injuries affecting the feet and lower limbs while optimizing biomechanics; the nutritionist, who oversees diet and recovery to support injury prevention and physical development; and family members, who serve as a source of support for adherence to prevention programs, emotional monitoring, and a safe return to competition.

Finally, in developmental settings, the systematic integration of psychological support strategies and interdisciplinary intervention may contribute not only to reducing the risk of new injuries, but also to promoting emotional well-being, sport adherence, and healthy long-term development in young soccer players.

## 7. Conclusions

The findings of the present study indicate that, in youth soccer players, a history of sports injuries is primarily associated with higher levels of anxiety, in both its trait and state dimensions. As the number of injuries increases, anxiety shows a progressive rise, reaching statistically significant differences between players with no injuries and those with a prior injury history, whereas stress and depression only showed nonsignificant upward trends.

Although no statistically significant differences were found in the distribution of injuries according to sex, playing position, or competitive category, descriptive patterns were identified that suggest possible variations associated with specific developmental stages and sport-related demands, although their interpretation is limited by the size of some subgroups.

Taken together, these findings reinforce the need to incorporate the psychological dimension, especially anxiety, into the prevention, assessment, and recovery of sports injuries in young soccer players, promoting a comprehensive approach to the injury process that goes beyond an exclusively physical perspective and integrates individual, contextual, and emotional factors. Future studies should employ longitudinal designs with larger samples and multiple clubs, while incorporating objective injury measures and longitudinal monitoring of mental health, in order to clarify the directionality of the relationship between injuries and anxiety, as well as the role of other contextual and personal variables.

## Figures and Tables

**Table 1:** Number of injuries by sex in young football players.

Sex	No Injuries	One–Two Injuries	More than Two Injuries	Total
Male (n = 79)	20 (25.3%)	44 (55.7%)	15 (19.0%)	79 (100%)
Female (n = 67)	19 (28.4%)	36 (53.7%)	12 (17.9%)	67 (100%)
Total (N = 146)	39 (26.7%)	80 (54.8%)	27 (18.5%)	146 (100%)

Note. N (total sample) and n (subgroups). χ^2^(2) = 0.17, *p* = 0.917. All expected frequencies were ≥5.

**Table 2:** Number of injuries by primary playing position in young football players.

Primary Playing Position	No Injuries	One–Two Injuries	More than Two Injuries	Total
Goalkeeper (n = 14)	1 (7.1%)	9 (64.3%)	4 (28.6%)	14 (100%)
Defender (n = 48)	16 (33.3%)	23 (47.9%)	9 (18.8%)	48 (100%)
Midfielder (n = 30)	6 (20.0%)	18 (60.0%)	6 (20.0%)	30 (100%)
Forward (n = 54)	16 (29.6%)	30 (55.6%)	8 (14.8%)	54 (100%)
Total (N = 146)	39 (26.7%)	80 (54.8%)	27 (18.5%)	146 (100%)

Note. N (total sample) and n (subgroups). χ^2^(6) = 5.47, *p* = 0.485. Two cells (16.7%) had expected frequencies < 5 (minimum expected frequency = 2.59).

**Table 3:** Number of injuries by competitive category in young football players.

Current Competition Category	No Injuries	One–Two Injuries	More than Two Injuries	Total
Infantil (n = 7)	5 (71.4%)	2 (28.6%)	0 (0.0%)	7 (100%)
Cadete (n = 28)	9 (32.1%)	13 (46.4%)	6 (21.4%)	28 (100%)
Juvenil (n = 94)	21 (22.3%)	53 (56.4%)	20 (21.3%)	94 (100%)
Senior (n = 17)	4 (23.5%)	12 (70.6%)	1 (5.9%)	17 (100%)
Total (N = 146)	39 (26.7%)	80 (54.8%)	27 (18.5%)	146 (100%)

Note. N (total sample) and n (subgroups). χ^2^(6) = 11.62, *p* = 0.071. Five cells (41.7%) had expected frequencies < 5 (minimum expected frequency = 1.29). This result should be interpreted with caution.

**Table 4:** Descriptive statistics of mental health indicators by number of injuries.

Variable	No Injuries (n = 39)	One–Two Injuries (n = 80)	More than Two Injuries (n = 27)	Total (N = 146)
STAI-T	M = 14.90, SD = 7.15	M = 18.96, SD = 9.76	M = 20.19, SD = 8.45	M = 18.10, SD = 9.06
Stress (DASS-21)	M = 4.28, SD = 3.47	M = 5.31, SD = 4.16	M = 6.30, SD = 4.20	M = 5.22, SD = 4.02
Anxiety (DASS-21)	M = 2.97, SD = 2.95	M = 4.12, SD = 3.90	M = 5.78, SD = 4.48	M = 4.12, SD = 3.88
Depression (DASS-21)	M = 2.85, SD = 3.28	M = 3.56, SD = 3.57	M = 4.15, SD = 4.73	M = 3.48, SD = 3.79

Note. N (total sample) and n (subgroups). STAI-T = Trait Anxiety Inventory; DASS-21 = Depression, Anxiety and Stress Scales. M = mean; SD = standard deviation. Values are presented according to number of injuries reported by participants.

**Table 5:** Post hoc comparisons (Games-Howell) of anxiety indicators according to number of injuries.

Variable	Comparison	MD	SE	CI 95%	*p* Adjusted
Trait anxiety	No injuries vs. 1–2 injuries	−2.87	1.32	[−5.48, −0.26]	0.038 *
	No injuries vs. >2 injuries	−3.31	1.72	[−6.71, 0.09]	0.056 †
	1–2 injuries vs. >2 injuries	−0.44	1.64	[−3.67, 2.79]	0.874
Anxiety (DASS-21)	No injuries vs. 1–2 injuries	−1.15	0.73	[−2.58, 0.28]	0.124
	No injuries vs. >2 injuries	−2.81	0.96	[−4.69, −0.93]	0.012 *
	1–2 injuries vs. >2 injuries	−1.66	0.94	[−3.50, 0.18]	0.087 †

Note. * *p* < 0.05, statistically significant difference; † *p* ≤ 0.10, statistical trend (trend toward significance); MD = mean difference; SE = standard error; CI = confidence interval; d = Cohen’s d. Adjusted *p* values were calculated using the Games–Howell procedure.

## Data Availability

The raw data supporting the conclusions of this article will be made available by the authors, without undue reservation.

## Abbreviations

The following abbreviations are used in this manuscript:

DASS-21	Depression Anxiety Stress Scales–21
STAI	State–Trait Anxiety Inventory
STAI-R	Trait Anxiety
